# Effects of *Artemisia argyi* Powder on Egg Quality, Antioxidant Capacity, and Intestinal Development of Roman Laying Hens

**DOI:** 10.3389/fphys.2022.902568

**Published:** 2022-08-25

**Authors:** Jiayi Chen, Fengming Chen, Simin Peng, Yangjiang Ou, Binsheng He, Yinghui Li, Qian Lin

**Affiliations:** ^1^ Academician Workstation, Hunan Key Laboratory of the Research and Development of Novel Pharmaceutical Preparations, Changsha Medical University, Changsha, China; ^2^ College of Animal Science and Technology, Hunan Agricultural University, Changsha, China; ^3^ Institute of Bast Fiber Crops, Chinese Academy of Agricultural Sciences, Changsha, China

**Keywords:** Artemisia argyi, laying hens, egg quality, antioxidative capacity, intestinal development

## Abstract

This study was conducted to evaluate the effect of dietary supplementation with *Artemisia argyi* (*A. argyi*) on egg quality, serum biochemical, antioxidant capacity, and intestinal development in Roman laying hens. A total of 432 (34-week-old) Roman hens were randomly divided into control group and three experimental groups. The control group was fed a basal diet, and the experimental group was fed a basal diet with 1%, 2%, and 3% *A. argyi* powder, respectively. The results showed that dietary supplementation of 2% *A. argyi* to the diet increased egg weight and egg white weight, and the daturic acid (C17:0), stearic acid (C18:0), eicosadienoic acid (C20:2), docosahexaenoic acid (C22:6n-3), α-linolenic acid (C18:3n-3), linoleic acid (C18:2n-6c), and polyunsaturated fatty acid (PUFA) in egg yolk. Meanwhile, the addition of 1∼3% *A. argyi* decreased serum urea. Moreover, dietary supplementation of 1% *A. argyi* promoted the antioxidative capacity of the hens by increasing hepatic T-SOD and CAT activities, as well as GSH-Px content. However, the addition of 3% *A. argyi* to the diet significantly increased the content of malondialdehyde in serum and liver and destroyed the intestinal morphology by increasing duodenal crypt depth. In conclusion, the addition level of *A. argyi* promoting egg quality and antioxidant capacity was at 2% and 1%, respectively.

## Introduction

*Artemisia argyi* (*A. argyi*) is a traditional Chinese herb with a history of use spanning over 2000 years. It is a perennial herb or small shrub with strong aroma. *Artemisia* belongs to the family Compositae, with more than 500 species (Abad et al., 2012). *A. argyi* contains a variety of bioactive chemicals such as polysaccharides, flavonoids, essential oils, and triterpenoids (Zhang et al., 2013). Increasing experiments have proved that *A. argyi* has many biological activities, such as antibacterial, anti-tumor, anti-oxidation, and immune regulation (Bao et al., 2013; Zhang et al., 2014). Some studies have suggested that the active components of *A. argyi* may exert anti-inflammatory effects through toll-like receptor 4 (TLR4)/nuclear factor-kappa B (NF-κB) signaling pathway (Zeng et al., 2014; Shin et al., 2017). As a plant homologous to medicine and food (Yamamoto et al., 2011; Li et al., 2018), owing to its antioxidant, antibacterial, hypoglycemic, hypolipidemic, and liver protection properties (Batiha et al., 2020), *A. argyi* can be applied in health care products and skin care products (Huang et al., 2012). Its own nutrients and bioactive ingredients gradually saw it applied in feed additives as well. Recently, studies have shown that *A. argyi* in diets can promote organic metabolism and improve animal digestion and absorption rate and production performance (Kim et al., 2012). Research indicated that 1% addition of *Artemisia* powder increased the weight gain of broilers by 217.9 g (Kaab et al., 2022). The addition of 2% *A. argyi* powder was reported to increase the activities of catalase (CAT) and glutathione peroxidase (GSH-Px) of the liver in broilers on d 21 as well as hepatic total superoxide dismutase (T-SOD) activity on d 42 and decrease hepatic malondialdehyde (MDA) content on d 42. Adding 1% *A. argyi* powder to the diet was superior for enhancing intestinal anti-oxidative capacity, represented by increased T-SOD activity of duodenum and total antioxidant capability (T-AOC) and CAT activity of jejunum, as well as decreased MDA in duodenum and ileum (Zhang et al., 2020a). When *A. argyi* extract was added to the diet, the optimal addition for improving intestinal antioxidant systems (SOD-CAT enzyme mechanism) of broilers at 21 d and 42 d was 0.5% and 1%, respectively (Zhao et al., 2016). It shows that *A. argyi* may have potential applications as antioxidants and production enhancers.

At present, there are more studies of *A. argyi* on broiler chickens, with relatively few focusing on laying hens. However, finding a natural feed additive which can effectively improve egg quality and release antioxidant stress contributes to the production of characteristic brand eggs, in line with people’s pursuit of healthy food. Therefore, the present study was conducted to investigate the effect of different levels of *A. argyi* on egg quality, serum biochemical indexes, antioxidant function, and intestinal mucosa morphological structure in Roman layers and its reasonable level of addition, so as to provide an experimental basis for the application of *A. argyi* in poultry production.

## Materials and Methods

### Preparation of *Artemisia argyi* powder

Fresh green *A. argyi* was collected from Hunan in July. Plant materials were washed with distilled water and dried in the shade at room temperature. The dry material was then cut into 1–2 cm pieces, crushed by a grinder, and sieved through an 80-mesh sieve to obtain the powder, which was then stored at ambient temperature (22–25°C) pending its use. The chemical constituents of A. argyi powder (analyzed value) are: genal energy 19.19 MJ/kg, dry matter 91.56%, crude protein 17.71%, crude fat 4.41%, crude ash 10.47%, crude fiber 16.64%, calcium 1.19%, and total phosphorus 0.28%.

### Animals and experimental details

A total of 432 (34-week-old) Roman hens were randomly divided into control group and three experimental groups with six replicates and 18 birds each. The control group was fed a basal diet, and the experimental group was fed a basal diet with 1%, 2%, and 3% *A. argyi* powder, respectively. The trial lasted for 8 weeks including the 2-week adaptation period. The basic diet was formulated to contain similar levels of CP and to meet recommendations of the National Research Council (NRC) for laying hens (1994), whose composition and nutritional level are shown in Table 1.

**TABLE 1:** Composition and nutrient levels of basal diets (air-dry basis, %).

Items	Diets			
	0.00%	1.00%	2.00%	3.00%
Ingredients				
Corn	57.57	55.49	53.32	51.19
Soybean meal	30.01	30.37	30.77	31.15
Artemisia argyi powder	0.00	1.00	2.00	3.00
Oil	0.97	1.69	2.46	3.21
Limestone	8.45	8.45	8.45	8.45
3% Premix^a^	3.00	3.00	3.00	3.00
Total	100.00	100.00	100.00	100.00
Nutrient levels^b^				
ME/(Mcal/kg)	2.75	2.75	2.75	2.75
Crude protein	17.00	17.00	17.00	17.00
Crude fiber	3.04	3.04	3.05	3.05
Calcium	3.50	3.50	3.50	3.50
Total phosphorus	0.54	0.53	0.53	0.52
Available phosphorus	0.32	0.32	0.32	0.32
Lysine	0.95	0.95	0.96	0.96
Methionine	0.36	0.36	0.36	0.36
Methionine + Cystine	0.65	0.65	0.65	0.64

a The premix provided the following (per kilogram of complete diet) micronutrients: VA, 6 000 IU, VD_3_ 2 500 IU, VE, 25 mg, VK_3_ 2.25 mg, VB_1_ 1.8 mg, VB_2_ 7 mg, VB_6_ 4 mg, VB_12_ 0.2 mg, *d* -pantothenic acid 12 mg, nicotinic acid 35 mg, biotin 0.14 mg, folic acid 0.8 mg, Cu (as copper sulfate) 11 mg, Zn (as zinc sulfate) 70 mg, Fe (as ferrous sulfate) 60 mg, Mn (as manganese sulfate) 115 mg, Se (as sodium selenite) 0.30 mg, and I (as potassium iodide) 0.4 mg.

b Nutrient levels are calculated values.

The experiment was carried out in two-layer full-step type chicken cages (1.2 m × 1.2 m × 0.5 m), with one cage for each repetition, and each group was evenly distributed in 1–2 layers. Chickens were fed twice a day (07:00 and 15:30) freely and drunk water through a nipple-type dispenser, and were provided with natural lighting and artificial lighting in the morning and evening to ensure the constant 16 h-lighting time every day. Infrared heating devices were used to maintain the temperature automatically. Humidity was controlled at about 65%. All eggs were collected on time at 12:00 and 17:00. The immunization procedure was carried out in accordance with the provisions of chicken farm epidemic prevention.

At the end of the experiment, blood samples were taken from a vein in the hen’s wing (two birds/replicate, 12 birds/treatment). The whole blood was coagulated in a test tube at room temperature and centrifuged at 3,500 rpm for 15 min. Serum samples were separated and stored at −20 °C until it was used to measure antioxidant and biochemical indicators. After blood collection, the hens were euthanized by carbon inhalation. After the abdominal cavity was opened, the tissues of small intestine (duodenum, jejunum, and ileum) were cut into 3–5 cm pieces and fixed in 10% neutral formalin solution. The liver tissue samples were taken at 2 g, kept in the centrifuge tube, and frozen at −20 °C for later analysis.

### Egg quality and yolk fatty acid measurements

The egg quality and yolk fatty acid was measured as previously described (Liu et al., 2021). Egg shape index was calculated as the ratio of the vertical and horizontal diameter of the egg. Eggshell strength was measured by the eggshell strength tester (EFR-01, Orka Co., Ltd.). Eggshell thickness (no shell membrane) was measured through the average values of three different sites (top, middle, and bottom of egg) using the eggshell thickness tester (NFN-380, Japan FHK Co., Ltd.). Haugh unit and yolk color were determined by SONOVA automatic egg quality analyzer (Orka Food Technology Ltd, Ramat Hasharon, Israel). After weighing the eggs on an electronic balance, the yolks and whites were separated and weighed to calculate the ratio of yolks and egg whites.

After been frozen, 5 g of egg yolk sample was put into the filter cartridge with 20 g sea sand. The filter cartridge was placed in the Sodel extractor and then refluxed in 65°C anhydrous ether bath until completely extracted. Then 3 ml of boron trifluoride-methanol was esteraped at 90°C for 7 min. The fatty acid content was detected using a HP-7890 gas chromatograph (HP, Uited States), a hydrogen flame ionization detector (FID), and a HPINNOWAX capillary column (30 m × 0.25 mm × 0.25μm).

### Serum biochemical indices

The contents of total protein (TP), glucose (Glu), albumin (ALB), globulin (GLB), and uric acid (UA) in serum were detected by Mindray BS-200 automatic biochemical analyzer (Shenzhen Mindray Biomedical Electronics Co., Ltd.) and kit (Nanjing Jiancheng Bioengineering Institute, Nanjing, China). The determination method was operated in strict accordance with the instructions of the kit.

### Antioxidant indices determination

T-SOD, T-AOC, GSH-Px, CAT, and MDA of the serum and liver were measured using commercial assay kits (Nanjing Jiancheng Bioengineering Institute, Nanjing, China) according to the manufacturer’s recommendations. The total protein content in the liver was determined by Coomassie brilliant blue method.

### Intestinal morphology analysis

Intestine samples (duodenum, jejunum, and ileum) were collected and fixed by 4% paraformaldehyde, dehydrated by alcohol, embedded by paraffin, and made into continuous sections for hematoxylin and eosin (H&E) staining. Then images were collected by Image-Pro Plus 4.5 software (Media Cybernetics Inc. Shanghai, China) for measuring the villus height and crypt depth to calculate the ratio of villus height to crypt depth.

### Statistical analysis

The test data were expressed as mean and standard error of mean (SEM). The four treatments means were compared by one-way analysis of variance (ANOVA) and tested by Duncan’s multiple range. *p* < 0.05 was regarded as statistically significant. Statistical model is as follows:

Y_ij_ = *μ*+T_i_ + e_ijk_, where, Y_ij_ means individual observed value, μ is the overall mean, T_i_ represents the treatmenI (i = 1, 2, 3, 4) effect, and e_ijk_ indicates a random error.

## Results

### The addition of *A. argyi* increased egg weight, egg white, and polyunsaturated fatty acid (PUFA) in yolk, especially 2% *A. argyi* addition

The effect of dietary *A. argyi* on egg parameters in laying hens is shown in Table 2. Inclusion of *A. argyi* in the diet had no effect on any egg parameters, except egg weight and egg white weight. The addition of 2% and 3% *A. argyi* to the diet significantly increased egg weight and reduced shell thickness (*p* < 0.05). In addition, 2% *A. argyi* in the diet significantly increased the egg white weight (*p* < 0.05).

**TABLE 2:** Effects of Artemisia argyi Meal on Fatty Acid Composition in Egg Yolk of Roman Laying Hens (%).

actItems	Artemisia argyi powder level				SEM	*p*-value
	0%	1%	2%	3%		
Average egg weight/g	57.51b	57.99b	63.23a	63.46a	0.88	<0.01
Shell strength/(kg/cm^2^)	4.59	5.04	4.82	4.86	0.12	0.66
Shell thickness/mm	0.38	0.37	0.37	0.36	0.00	0.41
Haugh unit	69.06	74.41	66.30	72.43	1.35	0.15
Shape index	1.33	1.34	1.31	1.32	0.01	0.36
Egg yolk color	4.38	4.50	4.20	4.00	0.09	0.20
Egg yolk weight/g	15.97	14.95	16.47	15.71	0.27	0.32
Egg white weight/g	35.60b	37.28b	49.03a	39.25b	1.56	<0.01
Egg yolk relative weight/%	28.44	25.77	25.99	25.82	0.43	0.07
Egg white relative weight/%	63.15	64.29	64.08	64.25	0.50	0.85

The effect of dietary *A. argyi* on fatty acid composition in egg yolk is shown in Table 3. The proportion of heptadecanoic acid (C17:0) (*p* < 0.01), eicosadienoic acid (C20:2) (*p* < 0.01), docosahexaenoic acid (C22:6n-3) (*p* < 0.01), α-linolenic acid (C18:3n-3) (*p* < 0.05), linoleic acid (C18:2n-6c) (*p* < 0.05), and polyunsaturated fatty acid (PUFA) (*p* < 0.05) significantly increased as the *A. argyi* addition in the diet increased from 0% to 2%, but when it increased to 3%, these acids no longer increased but decreased. The stearic acid (C18:0) proportion of 2% *A. argyi* addition group was higher than other three groups (*p* < 0.05). The C18:1n-9t proportion of 2% *A. argyi* addition was significantly higher than that of 1% and 3% *A. argyi* addition (*p* < 0.01). No obvious effect of diet was observed on other fatty acids (*p* > 0.05).

**TABLE 3:** Effects of Artemisia argyi Meal on Fatty Acid Composition in Egg Yolk of Roman Laying Hens (%).

Items	Artemisia argyi powder level				SEM	*p*-value
	0%	1%	2%	3%		
Saturated fatty acid (SFA), %						
C12:0	0.00	0.00	0.01	0.00	0.01	0.51
C14:0	0.34	0.34	0.33	0.28	0.04	0.37
C15:0	0.04	0.05	0.04	0.05	0.01	0.11
C16:0	25.60	24.77	24.40	24.39	1.21	0.67
C17:0	0.13c	0.16ab	0.17a	0.15bc	0.02	0.01
C18:0	8.57b	8.29b	9.62a	8.21b	0.60	0.01
C20:0	0.03	0.00	0.01	0.01	0.01	0.11
C22:0	0.05	0.05	0.05	0.02	0.02	0.08
Total SFA	34.71	33.55	31.32	33.08	0.64	0.28
Monounsaturated fatty acid (MUFA), %						
C14:1	0.09	0.08	0.07	0.05	0.03	0.61
C16:1	3.11	3.53	2.72	2.64	1.07	0.88
C20:1	0.23	0.20	0.21	0.21	0.02	0.61
C18:1n-9t	0.14ab	0.12bc	0.16a	0.10c	0.02	<0.01
C18:1n-9c	40.81	38.70	37.51	37.67	2.05	0.19
Total MUFA	43.34	41.46	40.61	40.66	0.86	0.70
Polyunsaturated fatty acid (PUFA), %						
C20:2	0.15c	0.19bc	0.23a	0.20b	0.03	<0.01
C22:6n-3	1.13c	1.34b	1.60a	1.43ab	0.20	<0.01
C18:3n-3	0.54c	0.66bc	0.97a	0.79ab	0.18	0.01
C18:3n-6	0.15	0.14	0.12	0.14	0.03	0.4
C18:2n-6c	14.84b	18.48ab	22.17a	20.45a	3.28	0.03
C20:3n-6	0.33	0.25	0.26	0.27	0.05	0.12
C20:4n-6	2.97	3.02	2.71	2.97	0.18	0.15
Total PUFA	20.12b	23.57ab	28.10a	26.24a	1.13	0.02

### The addition of *A. argyi* significantly decreased the amount of UA in serum

As shown in Table 4, inclusion of *A. argyi* in the diet had no apparent effect on GLU, TP, ALB, and GLB in serum (*p* > 0.05). However, the addition of *A. argyi* significantly decreased the amount of UA in serum (*p* < 0.05).

**TABLE 4:** Effects of Artemisia argyi on Serum Biochemical Indices of Roman Laying Hens.

Items	Artemisia argyi powder level				SEM	*p*-value
	0%	1%	2%	3%		
GLU (mmol/L)	12.04	12.60	10.53	11.47	0.56	0.76
TP (g/L)	72.44	72.39	72.85	77.79	2.24	0.91
ALB (g/L)	23.88	23.28	23.52	23.68	0.56	0.99
GLB (g/L)	52.97	54.97	52.55	53.32	2.08	0.98
UA (μmol/L)	93.99a	67.81b	66.97b	63.54b	4.99b	0.04

### The addition of 2% *A. argyi* enhanced the antioxidant capacity in body, but 3% *A. argyi* addition had the opposite effect

Table 5 demonstrated the effect of dietary *A. argyi* on serum antioxidant indices in laying hens. Composed to other three groups, the GSH-Px of 2% *A. argyi* addition in the diet was remarkably raised (*p* < 0.05). And for the group of 3% *A. argyi* addition, its serum GSH-Px was significantly lower than other three groups (*p* < 0.05), while its serum MDA was significantly higher than the other three groups (*p* < 0.05).

**TABLE 5:** Effects of Artemisia argyi on Serum Antioxidant Indices of Roman Laying Hens.

Items	Artemisia argyi powder level				SEM	*p*-value
	0%	1%	2%	3%		
T-AOC/(U/ml)	0.41	0.44	0.49	0.53	0.02	0.20
T-SOD/(U/ml)	22.73	26.50	44.37	37.87	4.00	0.22
GSH-Px/(U/ml)	282.04b	258.20b	389.27a	144.43c	33.14	<0.01
CAT/(U/ml)	15.51	13.91	14.39	14.69	0.39	0.56
MDA/(nmol/ml)	1.42b	1.42b	1.42b	2.99a	0.20	<0.01

### The addition of 1% *A. argyi* had the most beneficial impact on liver antioxidant indices, 2% addition secondly, 3% addition inversely

As shown in Table 6, the CAT amount in liver of 1% *A. argyi* addition (*p* < 0.05) group and the MDA amount in liver of 3% *A. argyi* addition group (*p* < 0.05) were the highest among the four groups, respectively. The T-SOD and GSH-Px amounts in liver of 3% *A. argyi* addition were both below those of 1% and 2% *A. argyi* addition (*p* < 0.05), and the GSH-Px of 0% *A. argyi* addition was also significantly lower than that of 1% *A. argyi* addition (*p* < 0.05).

**TABLE 6:** Effects of Artemisia argyi on liver Antioxidant Indices of Roman Laying Hens.

Items	Artemisia argyi powder level				SEM	*p*-value
	0%	1%	2%	3%		
T-AOC/(U/ml)	0.48	0.44	0.43	0.45	0.02	0.85
T-SOD/(U/g)	190.63ab	267.63a	265.63a	140.85b	19.71	0.05
GSH-Px/(U/g)	15.40bc	22.71a	20.22ab	11.75c	1.57	0.02
CAT/(U/g)	23.90b	31.95a	24.43b	23.93b	1.49	0.05
MDA/(nmol/g)	36.31b	45.31b	44.75b	79.01a	5.51	<0.01

### The addition of 3% *A. argyi* impaired the morphological structure of the intestine

**FIGURE 1:**
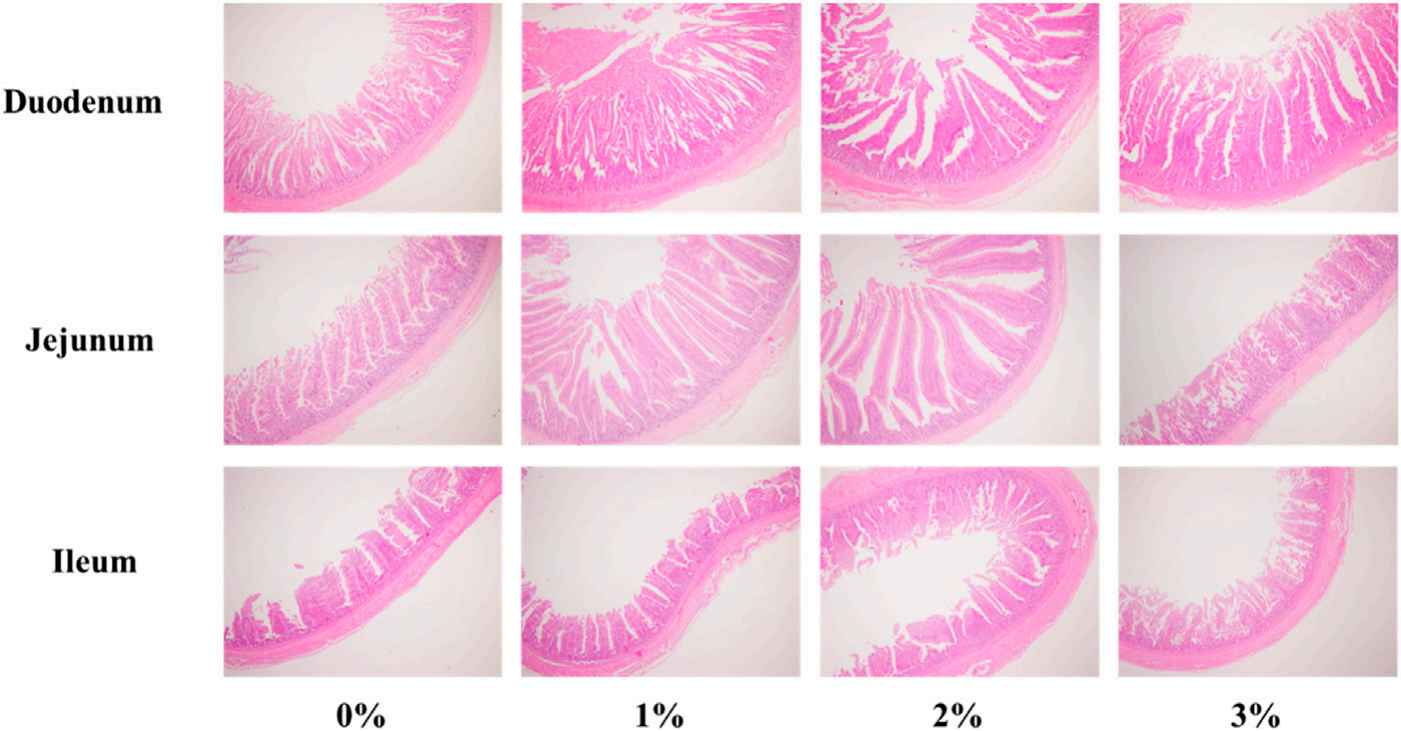
Effects of Artemisia argyi on intestinal morphology of duodenum, jejunum and ileum of Romance laying hens. HE 4 × 10.

Figure 1 showed the morphological structure of the intestine. As shown in Table 7, dietary *A. argyi* significantly increased crypt depth of duodenum in laying hens (*p* < 0.05). Composed to the addition of 1% and 2% *A. argyi*, villus height in jejunum of 3% *A. argyi* addition was significantly reduced (*p* < 0.05). In ileum, villus height of 2% *A. argyi* addition was significantly higher than that of 1% and 3% *A. argyi* addition (*p* < 0.05).

**TABLE 7:** Effects of Artemisia argyi on Intestinal Histological Morphology of Roman Laying Hens (μm).

Items	Artemisia argyi powder level				SEM	*p*-value
	0%	1%	2%	3%		
Duodenum						
Villus height	515.20	551.30	553.51	642.30	21.07	0.15
Crypt depth	57.61b	78.46a	72.79a	75.18a	3.30	0.05
Villus height/crypt depth	8.94	7.02	7.65	8.56	0.34	0.16
Jejunum						
Villus height	514.33ab	585.57a	575.49a	448.46b	22.12	0.03
Crypt depth	76.23	65.88	63.76	71.30	2.37	0.30
Villus height/crypt depth	6.76	6.95	9.02	6.29	0.53	0.37
Ileum						
Villus height	306.10ab	260.81b	383.62a	241.79b	21.19	0.05
Crypt depth	42.18	45.71	47.26	43.92	1.28	0.57
Villus height/crypt depth	7.26	5.70	8.11	5.68	0.43	0.92

## Discussion

Egg weight is a key indicator reflecting production performance of hens. Total egg weight is influenced by egg production rate, average egg weight, egg breakage rate, and other indicators, thus it is a comprehensive indicator reflecting the overall production performance of hens. The results of this experiment showed that the addition of 2% and 3% *A. argyi* to the diet increased egg weight and egg white weight to different degrees. Fatty acids are important lipid molecules in cells, which participate in many biological processes *in vivo*. Studies have shown that the dietary fatty acids composition is an important factor affecting human health, for instance, excessive intake of saturated fatty acids (SFAs) and cholesterol will cause inflammation and insulin resistance (Grundy, 1987), whereas PUFAs have positive biological functions. PUFAs are the main fatty acids in *A. argyi*, accounting for about 52.1%, followed by SFAs and monounsaturated fatty acids (MUFAs) (40.8% and 7.1% respectively) (Song et al., 2019). In the present study, additive 2% and 3% *A. argyi* significantly increased the eicosadienoic acid (C20:2), docosahexaenoic acid (C22:6n-3), α-linolenic acid (C18:3n-3), linoleic acid (C18:2n-6c), and PUFA contents in egg yolk, particularly the 2% group which increased the most. Kim et al. (2002) also found dietary wormwood increased omega-3 fatty acid contents of loin in beef cattle. According to Kim et al. (2015), linolenic acid (C18:3) is the most abundant fatty acid in *A. argyi*, with a relative percentage content of 36.36%. It was followed by palmitic acid (C16:0) and linoleic acid (C18:2) with 18.82% and 15.73%, respectively. The increased proportion of unsaturated fatty acids in egg yolks indicated the possibility that *A. argyi* favorably regulated the nutrient composition of egg yolks to some extent, pointing out a direction for subsequent studies to improve the fatty acid composition of eggs.

Early studies have shown that liver and kidney are the main organs of uric acid production in animals, and the production capacity of liver is higher than that of kidney in poultry (McFarland and Coon, 1984). UA is the end-product of amino acid metabolism in poultry, mainly generated from the degradation of proteins and nucleic acids and excreted by the kidneys. The UA content in serum reflects the balance of amino acids in diet and the level of protein catabolism in poultry kidney. Lower UA content means lower nitrogen excretion and more nitrogen deposition, which is beneficial in improving laying performance and protecting the liver and kidneys. In this experiment, the downward content of UA in each group after adding *A. argyi* indicated that *A. argyi* supplement can improve the utilization rate of protein and amino acids and promotes protein synthesis in laying hens, which may account for enhanced egg weight and egg white weight.

Redox homeostasis is of vital importance to cells, tissues, and organs of the body, and its maintenance depends mainly on the dynamic balance between oxidative and antioxidant systems. Once this dynamic balance is disrupted, oxidative stress occurs due to excessive reactive oxygen species (ROS) production or insufficient antioxidant capacity of the body (Yin et al., 2014). ROS can cause peroxidative damage to cell membrane lipids and produce MDA to attack polyunsaturated fatty acids in biological cell membranes, which can further trigger lipid peroxidation and lead to more severe oxidative stress damage (Shi et al., 2006). Therefore, MDA is often regarded as a marker of lipid oxidative damage. CAT and SOD play a crucial role in protecting the body from oxidative damage, with SOD converting ROS to H_2_O_2_ and CAT converting H_2_O_2_ to O_2_ and H_2_O (Finkel and Holbrook, 2000). GSH-Px has been found to scavenge superoxide and lipid hydroperoxide radicals (Arthur, 2001). The present study revealed that the addition of 1% of *A. argyi* to the diet increased hepatic CAT and GSH-Px content. Zhang et al. (2020a) also observed the same results in broiler chickens when adding 2% *A. argyi* powder to the diet. They also found T-SOD increased and MDA decreased in liver. This benefit might be attributed to the high antioxidant content of *A. argyi*, such as total phenolic compounds, which is positively associated with antioxidant activity (Song et al., 2019). Researchers found that the bioactive components (polysaccharides, flavonoids, and polyphenols) contained in *A. argyi* have a strong ability to scavenge free radicals (Lan et al., 2010; Melguizo-Melguizo et al., 2014; Han et al., 2017). Nevertheless, when the addition amount reached 3% in the present study, MDA levels in liver and serum were significantly increased while serum GSH-Px levels were significantly reduced. However, there was a report that 1% *A. argyi* extract supplementation tended to increase the activities of serum GSH-Px and CAT, but significantly reduced serum MDA level in broilers (Zhang et al., 2020b). These results illustrated that laying hens may have a greater sensitivity to *A. argyi* than broilers and a 1% addition helps to enhance the antioxidant function, but when increased to 3%, this function is impaired. The above results showed that the *A. argyi* addition of 1% is superior to 2% and 3% on improving the antioxidant capacity of laying hens.

The villi height and crypt depth of the intestine play an important role in nutrient absorption and providing a protective barrier (Liu et al., 2012). The villi is an important component of the intestinal tract, whose height reflects the absorption capacity of the small intestine (Pluske et al., 1997). Therefore, villi height, crypt depth, and the ratio of villi height to crypt depth are key indicators for assessing intestinal health and function (Liu et al., 2012). In the current trial, compared with the control group, the addition of 3% *A. argyi* to the diet significantly increased the crypt depth of the duodenum and tended to decrease the villus height of the jejunum and ileum. Chu and Song (2012) reported that more than 8% addition of wormwood decreased growth performance of rats on account of decreased nutrient digestibility. Thus, *A. argyi* seems to be potentially toxic. The results showed that when the addition of *A. argyi* reaches 3%, it destroys the morphological structure of the intestinal tract, which is not conducive to the absorption of nutrients.

## Conclusion

In conclusion, dietary supplementation with 2% *A. argyi* was beneficial for improving egg quality and increasing polyunsaturated fatty acids in egg yolk. As far as enhancing the antioxidant function, the 1% addition was better. The addition of 3% reduced the antioxidant capacity of the organism and damaged the morphological structure of the intestine.

## Data Availability

The original contributions presented in the study are included in the article/Supplementary Material, further inquiries can be directed to the corresponding author.
